# *In Vivo* Priming of Peritoneal Tumor-Reactive Lymphocytes With a Potent Oncolytic Virus for Adoptive Cell Therapy

**DOI:** 10.3389/fimmu.2021.610042

**Published:** 2021-02-18

**Authors:** Esther Giehl, Hiromichi Kosaka, Zuqiang Liu, Mathilde Feist, Udai S. Kammula, Michael T. Lotze, Congrong Ma, Zong Sheng Guo, David L. Bartlett

**Affiliations:** ^1^Departments of Surgery, University of Pittsburgh School of Medicine, and UPMC Hillman Cancer Center, Pittsburgh, PA, United States; ^2^Department of Visceral, Thoracic and Vascular Surgery, University Hospital Carl Gustav Carus, Dresden, Germany; ^3^Oncology Research Laboratories Oncology R&D Unit, Kyowa Kirin Co., Ltd., Shizuoka, Japan; ^4^Department of Surgery, CCM/CVK, Charité – Universitätsmedizin Berlin, Berlin, Germany

**Keywords:** oncolytic virus, CD8^+^ T cells, IL-15, adoptive cell therapy (ACT), solid tumor

## Abstract

Adoptive cell therapy (ACT) using autologous tumor infiltrating lymphocytes (TIL) achieves durable clinical benefit for patients from whom these cells can be derived in advanced metastatic melanoma but is limited in most solid tumors as a result of immune escape and exclusion. A tumor microenvironment (TME) priming strategy to improve the quantity and quality of TIL represents an important tactic to explore. Oncolytic viruses expressing immune stimulatory cytokines induce a potent inflammatory response that may enhance infiltration and activation of T cells. In this study, we examined the ability of an attenuated oncolytic vaccinia virus expressing IL15/IL15Rα (vvDD-IL15/Rα) to enhance recovery of lavage T cells in peritoneal carcinomatosis (PC). We found that intraperitoneal (IP) vvDD-IL15/Rα treatment of animals bearing PC resulted in a significant increase in cytotoxic function and memory formation in CD8^+^ T cells in peritoneal fluid. Using tetramers for vaccinia virus B8R antigen and tumor rejection antigen p15E, we found that the expanded population of peritoneal CD8^+^ T cells are specific for vaccinia or tumor with increased tumor-specificity over time, reinforced with viral clearance. Application of these vvDD-IL15/Rα induced CD8^+^ T cells in ACT of a lethal model of PC significantly increased survival. In addition, we found in patients with peritoneal metastases from various primary solid tumors that peritoneal T cells could be recovered but were exhausted with infrequent tumor-reactivity. If clinically translatable, vvDD-IL15/Rα *in vivo* priming would greatly expand the number of patients with advanced metastatic cancers responsive to T cell therapy.

## Introduction

Immunotherapy has emerged as one of the most promising, potentially curative new treatment approaches for patients with advanced metastatic cancer (1). Among several types of cancer immunotherapy, ACT and oncolytic viral immunotherapy both harbor great promise (2–4). Currently, ACT can take the form of CAR-T cell therapy (chimeric antigen receptor), TCR (T cell receptor) engineered T cell therapy, or TIL therapy (4–6). TIL therapy is personalized for the patient’s own tumor associated antigens and may limit tumor escape from immune mechanisms by allowing T cell responses to multiple tumor associated antigens (7). Primary limitations of TIL therapy include the inability to identify effective TIL from non-inflamed tumors, the exhausted phenotype of those expanded cells, their lack of suitable tumor specificity, and the immunosuppressive tumor microenvironment into which they are delivered. To date, durable responses in solid cancers have only been achieved in patients with cutaneous melanoma (6), uveal melanoma (8), cervical cancer (9), and a few other anecdotal selected tumor types. Many solid tumors are immune excluded, lack tumor-specific T cell infiltration, and are therefore not approachable by ACT (10). This limitation can potentially be overcome by local immune treatments that transform non-inflamed tumors and enhance the recovery of tumor-specific T cells for adoptive transfer (11).

The IL-15/IL-15 Rα complex exhibits a strong stimulatory effect on memory CD8^+^ T cells, enhancing their ability to self-renew and differentiate into more potent effector cells, rescale them ideal for ACT (12) particularly enhancing so-called tissue resident memory cells (13). Memory CD8^+^ T-lymphocytes harbor superior characteristics not only for expansion and differentiation *in vivo* but also enhanced antitumor effects following adoptive transfer (12). We tested here, using a replicating oncolytic virus (OV) that expresses IL-15/IL-15Rα (vvDD-IL15/Rα) *in vivo*, a strategy to enhance recovery of autologous tumor-specific memory CD8^+^ T cells which could be utilized for ACT.

OVs are attenuated viruses with tumor selective replication, enabling direct tumor cytotoxicity and promotion of anti-tumor immunity. Several oncolytic viruses have already been tested in clinical trials, with three of them reaching phase III trials (14). Vaccinia virus is a highly immunogenic oncolytic virus with efficient replication and a known safety profile in humans (15). The process of viral induced cell lysis, release of tumor associated antigens for delivery to antigen presenting cells, elicitation of specific damage associated molecular pattern (DAMP) molecules and subsequent toll like receptor (TLR) signaling, leads to a potent adaptive immune response (2). We and others have demonstrated that vaccinia virus expressing T cell cytokines increases anti-tumor T cell responses within the tumor microenvironment (TME) (16, 17). In addition to delivery of cytokines, oncolytic vaccinia virus has previously been designed to express nonsignaling CD19 protein. By tumor-selective surface expression of truncated B cell antigen CD19, oncolytic vaccinia virus promoted endogenous T cell infiltration in addition to *de novo* CAR T cell tumor control (18).

An IL-15 superagonist – IL-15 peptide fused with the alpha subunit of its receptor, IL15-Rα – is a promising immunotherapeutic agent for cancer treatment (19). In comparison to the native IL-15, the 20-fold prolonged biological half-life of the superagonist IL15/Rα further enhances proliferation of memory CD8^+^ T cells. Application of the superagonist peptide in mouse models of melanoma, pancreatic cancer, glioblastoma and lymphoma holds clear therapeutic benefits (20–23). Its therapeutic effect in solid and hematologic malignancies is under investigation in several phase I/II clinical trials (24, 25). We have previously shown that vvDD-IL15/Rα extends the survival of mice bearing colon and ovarian cancers, eliciting potent adaptive antitumor immunity. This is dependent on CD8^+^ T cells and to a lesser extent on NK and CD4^+^ T cells. Memory T cells protected against tumor recurrence *in vivo* in tumor re-challenge experiments following vvDD-IL15/Rα treatment (17).

With such a profound impact on the TME mediated by OVs, we hypothesized that local infection with a vaccinia virus expressing the IL-15 superagonist (IL-15/IL-15Rα) would lead to enhanced presence of tumor-specific T cells that could be recovered for adoptive transfer, advancing the therapeutic role of ACT for solid tumors. We explored the feasibility of intraperitoneal (IP) delivery of vvDD-IL15/Rα to promote peritoneal memory, tumor-antigen-specific T cells with enhanced anti-tumor effector function, suitable for ACT in murine syngeneic and immunocompetent models of colorectal and pancreatic cancers. As MC38 cancer cells express an endogenous retroviral tumor associated antigen (p15E) (26), we were able to use this model to further discriminate the anti-viral from the anti-cancer T cell function induced by infection with vvDD-IL15/Rα in the course of time. To explore the potential translation of our preclinical findings to human cancers, we have also characterized peritoneal CD4^+^ and CD8^+^ T cells from 14 patients with peritoneal tumors undergoing surgical excision and examined these T cells for memory and functional phenotype and anti-tumor response.

## Methods and Materials

*Tumor Cell Lines and Viruses*. Murine cancer cell lines, MC38 and CT26 colon cancer, and Panc02 pancreatic cancer were cultured in an incubator at 37°C with 5% CO_2_ and grown in DMEM supplemented with 1× penicillin and streptomycin (Invitrogen, Carlsbad, CA), 2 mM L-glutamine, and 10% fetal bovine serum (FBS) as previously reported (17). OVs vvDD and vvDD-IL15/Rα were generated, expanded, and purified in our laboratory as previously described (17).

*Murine Tumor Models and Treatment Regimens*. All animal studies were approved by the Institutional Animal Care and Use Committee of the University of Pittsburgh. Female seven- to eight-week-old immuncompetent C57BL/6J (B6; H-2K^b^) or BALB/c mice were purchased from the Jackson Laboratory (Bar Harbor, ME) and housed in specific pathogen-free conditions. For peritoneal carcinomatosis models, mice were injected intraperitoneal (IP) with 5.0x10^5^ MC38-luc or 2.5x10^5^ CT26-luc or 1.0x10^6^ Panc02-luc cancer cells, and monitored for tumor growth *via* bioluminescence imaging using the Xenogen IVIS Optical *In Vivo* Imaging System (Caliper Life Sciences, Hopkinton, MA) as described previously (16). Mice were subsequently randomized based on tumor growth and injected IP with 200 μl PBS, oncolytic vvDD, or vvDD-IL15/Rα at 5.0 x 10^7^ pfu/200 μl.

Peritoneal T cells were extracted from the peritoneal cavity *via* IP lavage (17). An 18G needle was inserted in the peritoneal cavity, 2–3 ml of 2%FBS-enriched PBS injected and aspirated repeatedly till a lavage volume of 12 ml was reached. Lavage fluid was strained over a 100 μM cell strainer, and red blood cells were lysed using ACK Lysing Buffer (Thermo Fisher Scientific, Waltham, MA) prior to final straining over a 40 μM cell strainer.

Tumor-infiltrating lymphocytes (TIL) were derived from collected tumors of the peritoneal cavity according to quoted days after OV treatment. After weighing, tissues were incubated in RPMI-1640 medium containing 2%FBS, 1 mg/ml collagenase IV (Sigma:#C5138), 0.1mg hyaluronidase (Sigma:#H6254) and 200U DNaseI (Sigma:#D5025) at 37°C for 1-2h to dissolve in single cells.

*ELISA Assay*. All visible tumor infestation was recovered from the peritoneal cavity according to stated days after vvDD, vvDD-IL15/Rα or PBS treatment, homogenized using Precellys 24 tissue homogenizer (Bertin Instruments, Rockville, MD) and supernatant extracted for further analysis. The concentration of IL15-IL15Rα fusion protein in tumor tissue supernatant was quantified using mouse IL-15 DuoSet ELISA kit (R&D Systems, #DY447-05) and IFN-γ concentrations were quantified with the ELISA MAX™ Standard Set Mouse IFN-γ (BioLegend, # 430801) according to the manufacturer’s instructions supplemented by protease inhibitor (Sigma Aldrich, #11697498001).

*In vitro Coculture Assays*. Lavage CD8^+^ T cells were separated from total isolated IP cells by using α-mouse CD8 microbead isolation protocol (Miltenl Biotec, San Diego, CA). Purified CD8^+^ T cells (1.0 x 10^5^) were co-cultured in RPMI 1640 with L-glutamine, 100 IE/ml Penicillin and 50 μg/ml Streptomycin at 37°C, 5% CO_2_ in 96-well plates with either 1.0 x 10^5^ 1500-rad irradiated splenocytes from B6 mice, 2.0 x 10^4^ 20000-rad irradiated MC38, Panc02, CT26 or 4T1 cells or 2.0 x 10^4^ 20000-rad irradiated and vvDD-infected Panc02 or 4T1 cells. Panc02 and 4T1 cells were preincubated with IFN-γ for 48 h before assay assembly to induce MHC class I expression. To test CD8^+^ T cell’s anti-viral activity, 1.0 x 10^6^ cells Panc02 or 4T1 cells were infected *in vitro* with 1e6 pfu vvDD 48 h before assay assembly. For epitope assays, stimulators were generated by incubating fresh splenocytes (isolated from B6 mouse) in the presence of 10^-7^ M concentrations of peptide for 45 min, then washed three times and irradiated (2500 cGy).

Human peritoneal T cell populations were purified according to CD3 MicroBeads, human protocol from Miltenyi Biotec from patient’s lavage samples to then be applied to coculture assays. 1.0 x 10^5^ CD3^+^ T cells were cocultured with purified CD14^+^ monocytes or 25,000-rad irradiated tumor digest in a 1:2 ratio. CD14^+^ monocytes were purified from PBMC according to CD14 MicroBeads, human protocol from Miltenyi Biotec.

*IFN-γ ELISpot Assay*. Following coculture, plates were incubated with biotinylated mouse IFN-γ mAb R4-GA2-Biotin, Mabtech, Cincinnati, OH). ELISpot plates were then developed according to vendor protocols for Vectastain Elite ABC and AEC Peroxidase substrate (SK-4200) kits (Vector Laboratories, Burlingame, CA) and analyzed using an ImmunoSpot analyzer (Cellular Technology, Shaker Heights, OH).

*RT-qPCR*. Total RNA was abstracted from tumor tissue using the RNeasy Kit (Qiagen, Valencia, CA) and used for cDNA creation. Quantitative PCR was performed with 50 ng of subsequent cDNA by TaqMan analysis on the StepOnePlus system (Life Technologies, Grand Island, NY) and relative gene expression calculated according to published protocols (16). All PCR primers were purchased from Thermo Fisher Scientific (Waltham, MA). Customized primers are as follows: murine p15E forward primer – GTACGGGATAGCATGGCCAAACTTAGAGAA; murine p15E reverse primer – CTACCGAAATCCTGTCTTTGATAAACTG. The gene expression was normalized to the housekeeping genes HPRT1 and expressed as fold increase (2^−ΔCT^), where ΔCT = CT _(Target gene)_ – CT _(HPRT1)_.

*Adoptive transfer therapy models*. Peritoneal CD8^+^ T cells were isolated from MC38 tumor-bearing mice 19 days after inoculation of 5.0 x 10^5^ MC38-luc cells and 9 days after treatment with 5.0e7 pfu vvDD or vvDD-IL15/Rα or an equal amount of PBS. Recipient MC38 tumor-bearing C57BL/6 mice were inoculated with 5 x 10^5^ MC38-luc cells 10 days prior to ACT. 12 h before ACT recipient mice were irradiated with 5 Gy. 3.5 x 10^5^ CD8^+^ T cells pooled per group were combined with 50.000 IU human IL-2 and transferred IP. Recipient mice received a total of 6 doses of 10^5^ IU IL-2 every 12 h beginning with ACT.

*Study population of human cancer patients*. Between January 2018 and October 2019 at UPMC Hillman Cancer Center, Pittsburgh, USA, body fluids including peritoneal fluid, matched blood and tumor samples were obtained in a prospective, nonselective fashion from 14 patients with peritoneal carcinomatosis prior to cytoreductive surgery under an IRB approved protocol (UPCI#17-220). Clinicopathologic information was obtained by reviewing electronic medical records (EMR) of patients.

*Isolation of PBMC or Lymphocyte in Malignant Fluid and Tumor Tissue*. Peripheral blood mononuclear cells (PBMCs) were isolated from 30 ml peripheral blood collected into heparinized tubes. Peritoneal lymphocytes were extracted from 1.0 liter of peritoneal lavage per patient. Lymphocytes were purified from PBMC or lavage samples by gradient centrifugation using Ficoll-Paque™ PLUS (GE Healthcare, Sweden) according to the manufacturer’s protocol. Single-cell suspensions from human tumors were generated with the Tumor Dissociation Kit, human (Miltenyi Biotec, #130-095-929) in association with gentleMACS Octo Dissociator with Heaters (#130-096-427).

*Flow Cytometry and Antibodies*. BUV395 conjugated anti-mouse CD3 (clone 145-2C11), BUV395 conjugated anti-mouse CD137 (clone 1AH2), BUV737 conjugated anti-mouse CD3 (clone 145-2C11), BUV395 conjugated anti-human CD8 (clone RPA-T8) were obtained from BD Biosciences. BV421 conjugated anti-mouse TIM-3 (clone RMT3-23), Zombie aqua, BV785 conjugated anti-mouse PD-1 (clone 29F.1A12), FITC conjugated anti-mouse CD103 (clone 2E7), PerCP-Cy5.5 conjugated anti-mouse CD62L (clone MEL-14), PE conjugated anti-mouse CD183 (clone CXCR3-173), APC conjugated anti-mouse CD122 (clone TM-ß1), Alexa Fluor 700 conjugated anti-mouse CD44 (clone IM7), APC-Cy7 conjugated anti-mouse CD8b, PE-Dazzle 594 conjugated anti-human CD3 (clone UCHT1), APC conjugated anti-human CD137 (clone 4B4-1), BV785 conjugated anti-human TIM-3 (clone F38-2E2), PE conjugated anti-human PD-1 (clone EH12.2H7), BV421 conjugated anti-human CCR7 (clone G043H7), APC-Cy7 conjugated anti-human CD134 (clone Ber-ACT35), AF700 conjugated anti-human CD45RO (clone UCHL1), PerCP-Cy5.5 conjugated anti-human CD4 (clone RPA-T4) were obtained from BioLegend. FITC conjugated anti-mouse CD8a (clone KT15) was purchased from Thermo Fisher Scientific. PE conjugated anti-mouse B8R TSYKFESV (Tetramer) (clone H-2Kb) and APC conjugated anti-mouse p15E KSPWFTTL (Tetramer) (clone H-2Kb) were obtained from MBL International. FITC conjugated anti-human CD122 (clone REA167) was purchased from Miltenyi Biotec.

Staining was performed according to manufacturer’s protocols. For intracellular staining, protein transport inhibitor containing monensin (BD GolgiStop™) and Fixation and Permeabilization buffer (eBioscience, Thermo Fisher Scientific) were applied according to manufacturer’s protocols. All samples were applied to LSRII or Fortessa FACS (BD Biosciences) and analyzed by using Flowjo software (Tree star).

*Statistical Analyses*. GraphPad Prism version 6 or 8 (GraphPad Software, Inc., San Diego, CA) analysis was performed using non-parametric Student’s t test and ordinary one-way ANOVA or two-way ANOVA for multiple comparisons. The overall survival (OS) was analyzed by Kaplan-Meier survival curves using the log-rank (Mantel-Cox) test. The standardized symbols were used in the figures, as follows: * p < 0.05; ** p < 0.01; *** p < 0.001; **** p < 0.0001; and NS: not significant.

## Results

### IL-15/IL-15Rα Enhances the Cytotoxic T Cell Response

**Figure 1:**
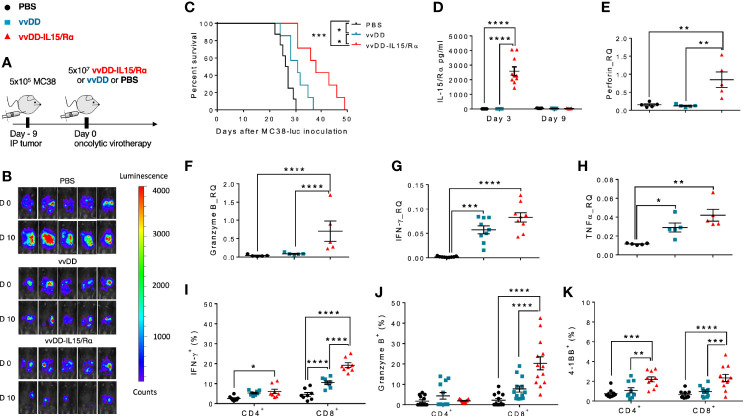
Oncolytic virus-based vaccinia virus expressing IL15/IL15Rα (vvDD-IL15/Rα) expression leads to enhanced cytotoxic T cell activation. **(A)** Experimental design: Immunocompetent B6 mice were inoculated IP with 5.0x10^5^ MC38-luc cells (day −9), randomized and treated with intraperitoneal (IP) injection of PBS, vvDD or vvDD-IL15/Rα at a dose of 5.0x10^7^ pfu/mouse (day 0). **(B)** Representative *in vivo* bioluminescence pictures of tumor burden on day 0 and day 10 following treatment. **(C)** vvDD-IL15/Rα prolongs survival in lethal peritoneal carcinomatosis with high tumor burden. **(D)** Intratumoral mouse IL15/IL15-Rα-complex was elevated 3 days following IP injection of vvDD-IL15/Rα. **(E–H)** Three days after IP therapy, mRNA expression in tumor tissue by qPCR analysis showed elevated levels of cytotoxic proteins, perforin **(E)** and granzyme B **(F)** after vvDD-IL15/Rα treatment. Levels of Th1 cytokines, IFN-γ **(G)** and TNF-α **(H)**, were increased in response to virus treatments relative to PBS. **(I–K)**: Lavage CD8^+^ T cells were isolated and directly analyzed *via* flow cytometry 3 days following treatment. **(I)** IFN-γ showed highest intracellular abundance in CD8^+^ T cells derived from vvDD-IL15/Rα treated animals. **(J)** Intracellular expression of granzyme B was significantly increased in lavage CD8^+^ T cells after vvDD-IL15/Rα injection. **(K)** The costimulatory molecule CD137 (4-1BB) showed increased surface expression in lavage CD4^+^ and CD8^+^ T cells in the vvDD-IL15/Rα treatment group. N=14. All values presented as mean ± SEM. *p < 0.05. **p < 0.01. ***p < 0.001. ****p < 0.0001. Data are combined from two independent experiments.

Immunocompetent C57BL/6 mice were injected with 5.0x10^5^ MC38-luc cancer cells into the peritoneal cavity. 9 days later, tumor-bearing mice were treated with 5.0x10^7^ pfu of vvDD, vvDD-IL15/Rα or PBS according to *in vivo* bioluminescence imaging (**Figure 1A**). Tumor imaging and animal survival confirmed our previous work (17), demonstrating an anti-tumor effect mediated by vvDD-IL15/Rα administration (**Figures 1B, C**). Three days following initial vvDD-IL15/Rα infection, IL-15/IL-15Rα levels rose to a detectable mean concentration of 2,581 pg/ml per 500 mg of tumor tissue by ELISA, while concentrations in tumors of PBS and vvDD treated mice were undetectable (**Figure 1D**). On day 3, high IL-15/IL-15Rα concentrations coincided with increased mRNA levels of cytotoxic proteins perforin and granzyme B in vvDD-IL15/Rα treated tumors, suggesting activated granule exocytosis from immune cells (**Figures 1E, F**). We also found increased mRNA expression of Th1 cytokines IFN-γ and TNF-α following treatment with either vvDD or vvDD-IL15/Rα compared to PBS treated controls, consistent with a cell-mediated inflammation to intracellular virus (**Figures 1G, H**). Furthermore, vaccinia virus treatment induced mRNA expression of the inhibitory immune receptors PD-1 (PDCD1 gene), PD-L1 and CTLA-4 enabling immune evasion when compared with PBS controls (**Supplemental Figures 1A–C**). Correlational analysis with flow cytometry showed that vvDD-IL15/Rα treatment resulted in higher percentages of CD8^+^ T cells expressing IFN-γ, granzyme B, and 4-1BB and of CD4^+^ cells expressing 4-1BB compared to vvDD or PBS treatment (**Figures 1I–K**). The IL-15/IL-15Rα returned to undetectable levels 9 days following vvDD-IL15/Rα treatment, suggesting rapid immunologic clearance of the virus (**Figure 1D**). Vaccinia virus A34R gene expression, a marker of vaccinia virus accumulation, further emphasizes this concept with vvDD and vvDD-IL15/Rα replication on day 3 and partial vvDD replication on day 6 following i.p. treatment, with undetectable levels on day 9 (**Supplemental Figure 1D**). In summary, these results indicate that high IL-15/IL-15Rα tumor abundance three days following vvDD-IL15/Rα treatment correlates with a cytotoxic CD8^+^ T cell immune response.

### vvDD-IL15/Rα Treatment Enriches an Activated and Memory CD8^+^ T Population Favorable for ACT

**Figure 2:**
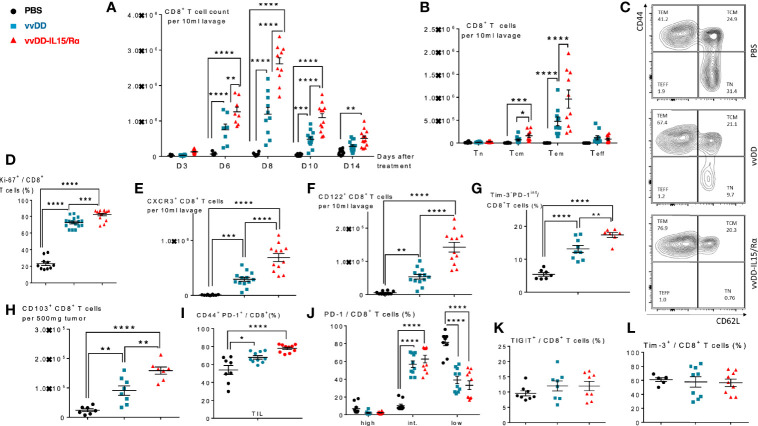
Vaccinia virus expressing IL15/IL15Rα (vvDD-IL15/Rα) treatment leads to enrichment of memory CD8^+^ T cells with an activated phenotype. Immunocompetent B6 mice were inoculated IP with 5.0x10^5^ MC38-luc cells (day −9), randomized and treated with intraperitoneal (IP) injection of PBS, vvDD or vvDD-IL15/Rα at a dose of 5.0x107 pfu/mouse (day 0). Ten days following i.p. oncolytic virotherapy, peritoneal CD8^+^ T cells were isolated *via* peritoneal lavage and characterized *via* flow cytometry (in addition to day 3, 6, 8, and 14 for figure A). **(A)** Regional delivery of vvDD-IL15/Rα greatly enhanced and prolonged peritoneal CD8^+^ T cell infiltration on day 6 to 14 following virotherapy. **(B)** The amount of peritoneal central memory (Tcm, CD44^+^ CD62L^+^) and effector memory (Tem, CD44^+^ CD62L^-^) T cells is increased 10 days following vvDD-IL15/Rα injection. **(C)** Representative flow cytometry charts of memory CD8^+^ T cell subsets. vvDD-IL15Rα treatment resulted in increased Ki67 **(D)**, CXCR3 **(E)**, CD122 **(F)**, Tim3^-^PD1^int^ **(G)**, CD103 **(H)**, and CD44^+^PD-1^+^ **(I)** staining compared to vvDD or PBS 10 days following injection. PD-1^high^ **(J)**, TIGIT^+^ **(K)**, and Tim-3^+^ **(L)** staining were not increased after vvDD-IL15/Rα treatment compared to vvDD or PBS treatment. Representative flow cytometry charts of PD-1 expression by treatment group are depicted in **Supplemental Figure 2**. N=8–12. All values presented as mean ± SEM. *p < 0.05. **p < 0.01. ***p < 0.001. ****p < 0.0001. Data are combined from two independent experiments.

In the same treatment model of lethal peritoneal colon cancers, we conducted a time course experiment, evaluating peritoneal CD8^+^ T cell expansion in response to viral treatment (**Figure 2A**). A significant increase in the number of purified CD8^+^ T cells was first detected on day 6 with a mean count of ~8.0x10^5^ and ~1.2 x 10^6^ CD8^+^ T cells in vvDD and vvDD-IL15/Rα treated animals, respectively. Mean lavage CD8^+^ T cell counts reached a peak value on day 8 following treatment with ~1.2x10^6^ and ~2.8 x 10^6^ CD8^+^ T cells following vvDD and vvDD-IL15/Rα treatment, respectively. Following this peak, CD8^+^ T cell counts declined in both VV-treated groups through day 10 and day 14, approaching near baseline CD8^+^ T cell values with ~3x10^5^ and ~6 x10^5^ total cells on day 14 in the vvDD and vvDD-IL15/Rα groups, respectively. PBS control derived CD8^+^ T cells marginally increased, coinciding with progressive malignant hemorrhagic ascites.

We next examined the CD8^+^ T cell phenotype at a time after viral clearance (17), when T cells can be safely harvested. On day 10 following treatment, the total numbers of peritoneal central memory CD8^+^ T cells (Tcm, CD44^+^ CD62L^+^) and effector memory CD8^+^ T cells (Tem, CD44^+^ CD62L^-^) were significantly increased following vvDD-IL15/Rα treatment (**Figures 2B, C**). vvDD-IL15/Rα derived CD8^+^ T cells had higher expression of Ki-67, CXCR3/CD183 and CD122 compared to vvDD or PBS controls, consistent with an activated phenotype (**Figures 2D–F**). An increased number of non-exhausted, activated Tim3^-^PD1^int^ CD8^+^ T cells was observed following vvDD-IL15/R treatment (**Figure 2G**). CD8^+^ TIL expressed significantly more CD103, consistent with an increase in tissue resident memory T cells (**Figure 2H**) and were more likely to show an antigen-experienced phenotype (CD44^+^ PD-1^+^) (**Figure 2I**) (27). The CD8^+^ T cells were more likely to express intermediate PD-1 levels after vaccinia treatment (**Figure 2J**, see **Supplemental Figures 2D–F** for representative flow cytometry charts), while other exhaustion markers (TIGIT and Tim-3) were unaltered in response to vaccinia or IL-15/IL-15Rα treatment (**Figures 2K, L**).

### vvDD-IL15/Rα Treatment Induces Endogenous Retroviral Tumor Associated Antigen p15E-Specific CD8^+^ T Cells

**Figure 3:**
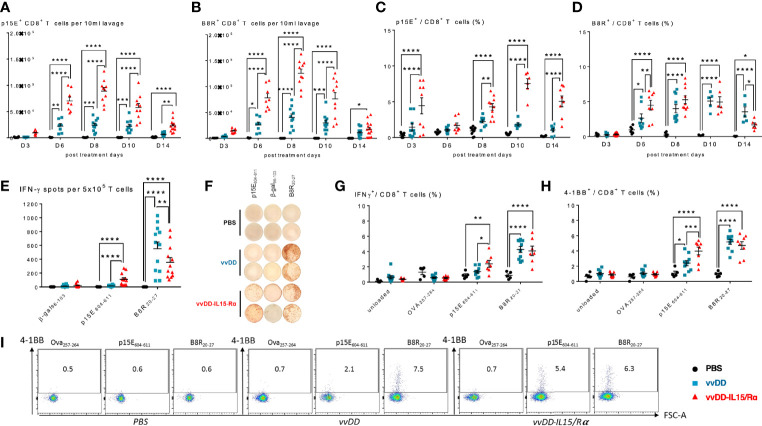
Vaccinia virus expressing IL15/IL15Rα (vvDD-IL15/Rα) treatment enhances CD8^+^ T cell reactivity to endogenous retroviral tumor-rejection antigen, p15E. 3, 6, 8, 10, and 14 days following i.p. oncolytic virotherapy with vvDD-IL15/Rα of late-stage MC38 tumor-bearing mice, peritoneal CD8^+^ T cells were analyzed by FACS using p15E **(A, C)** and B8R **(B, D)** tetramers. **(A)** Time course of p15E tetramer staining shows a marked increase in the number of peritoneal p15E Tetramer^+^ CD8^+^ T cells after vvDD-IL15/Rα treatment. **(B)** Time course of B8R staining shows a similar pattern, with slightly higher peak values. **(C, D)** When comparing the percentages of peritoneal p15E Tetramer^+^ CD8^+^ T cells, only vvDD-IL15/Rα induced a significant rise in comparison to PBS and vvDD treatment. The percentage of B8R Tetramer^+^ peritoneal CD8^+^ T cells per total peritoneal CD8^+^ T cells increases in response to vvDD and vvDD-IL15/Rα. **(E–I)** To validate the results from the tetramer experiments, we performed *in vitro* functional assays. Peritoneal CD8^+^ T cells, 10 days post treatment, were co-cultured with p15E_605-611_-, B8R_20-27_-, OVA_257-264_-, β-gal_96-103_-loaded, or unloaded irradiated splenocytes for 24 h and analyzed *via* IFN-γ-ELISPOT **(E, F)** or IFN-γ^+^ CD8^+^ T cells **(G)** and CD137(4-1BB)^+^ CD8^+^ T cells **(H, I)** *via* flow cytometry. All data represent the results of two independent experiments. All values presented as mean ± SEM. *p < 0.05. **p < 0.01. ***p < 0.001. ****p < 0.0001.

Next, using tetramers for a tumor-rejection, endogenous retroviral antigen, p15E, and an immunodominant vaccinia antigen, B8R, we examined the time course of CD8^+^ T cell receptor specificity following oncolytic virotherapy (**Figures 3A–D**). We found an increase in both p15E- and B8R-specific T cells in response to vvDD-IL15/Rα treatment with a peak in total reactive CD8^+^ cells against both antigens demonstrated on day 8 (**Figures 3A, B**). Correlating with viral clearance, vvDD-IL15/Rα leads to an increase in the percentage of p15E-specific CD8^+^ T cells on day 10 and 14 compared to stable or decreased B8R-specific cells (**Figures 3C, D**).

To further evaluate p15E-specificity following viral extinction, peritoneal CD8^+^ T cells were stimulated *in vitro* with the p15E_604-611_ peptide, B8R_20-27_ peptide, or control (β-gal_96-103_ or Ova_257-264_) peptide 10 days following treatment. As expected, vvDD-IL15/Rα and parental vvDD virus increased B8R-specific T cells. In comparison, only vvDD-IL15/Rα treatment increased p15E-specific T cells as measured by elevated intracellular IFN-γ or cell surface 4-1BB expression by flow cytometry (**Figures 3E–I**). In fact, vvDD-IL15/Rα led to decreased B8R-specific IFN-γ spots by ELISPOT compared with vvDD, while simultaneously increasing p15E-specific T cells (**Figures 3E, F**).

### Enhanced Anti-Tumor Reactivity of vvDD-IL15/Rα-Induced CD8^+^ T Cells Results in Improved Survival After ACT

**Figure 4:**
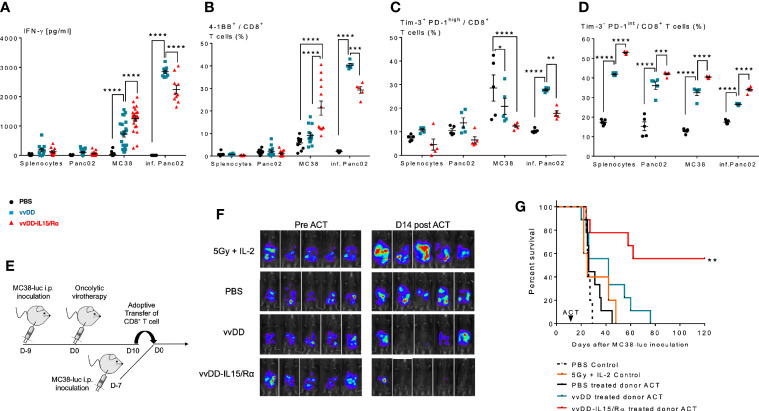
Enhanced anti-tumor reactivity of lavage CD8^+^ T cells following vaccinia virus expressing IL15/IL15Rα (vvDD-IL15/Rα) treatment correlates with improved survival after adoptive cell therapy (ACT). 10 days following i.p. oncolytic virotherapy with vvDD-IL15/Rα of late-stage MC38 tumor-bearing mice, reactivity of peritoneal CD8^+^ T cells was examined *in vivo* and *in vitro* 10 days following treatments. **(A–C)**: MC38- and vvDD-specific reactivity was measured after co-culture for 24 h *in vitro*. **(A)** vvDD-IL15/Rα treatment increased MC38 reactivity as measured by IFN-γ production; and **(B)** 4-1BB^+^ staining. **(C)** The percent of exhausted cells (Tim-3^+^ PD-1^high^) were decreased after co-culture with MC38 in response to vvDD-IL15/Rα treatment. **(D)** A higher percentage of the Tim-3^-^ PD-1^intermediate^ stained cells were present after vvDD-IL15/Rα in all co-culture conditions. **(E)** In ACT experiments, acceptor mice received 5.0x10^5^ MC38-luc cells 7 days prior to ACT and a preparatory regimen of sub-lethal whole-body irradiation (5 Gy) 12 h prior to ACT. 3,5x10^5^ total CD8^+^ T cells pooled per group were transferred IP, followed by IL-2 IP stimulation every 12 h six times. **(F)** Representative images of intraperitoneal (IP) tumor burden *via in vivo* bioluminescent imaging are displayed. Pictures were taken before adoptive transfer (D0), and 14 days post adoptive transfer (D14). **(G)** vvDD-IL15/Rα induced TIL increased long term survival of tumor-bearing mice as displayed by Kaplan-Meier survival curves. All data represent the results of two independent experiments with a minimum of 5 mice per treatment group, respectively. All values presented as mean ± SEM. *p < 0.05. **p < 0.01. ***p < 0.001. ****p < 0.0001.

First, we evaluated the ability of the peritoneal CD8^+^ T cells to recognize autologous MC38 tumor cells *in vitro*. Autologous splenocytes and an irrelevant B6 murine tumor, Panc02 served as negative control targets and vvDD-infected Panc02 cells as a measure of vaccinia virus T cell reactivity. Comparison of supernatant IFN-γ levels and cell surface 4-1BB expression demonstrated that vvDD-IL15/Rα therapy resulted in higher MC38 reactivity from CD8^+^ lavage cells compared with vvDD or PBS treatment. Interestingly, vvDD-IL15/Rα therapy led to lower vaccinia virus reactivity compared to vvDD (**Figures 4A, B**). Co-expression analysis of immunosuppressive Tim-3 and PD-1 on CD8^+^ T cells showed that terminally exhausted Tim-3^+^PD-1^hi^ CD8^+^ T cells were decreased following vaccinia infection (**Figure 4C**), while the Tim-3^-^ PD-1^int^ CD8^+^ T cells were increased following vvDD-IL15/Rα treatment in all coculture conditions (**Figure 4D**).

Secondly, peritoneal CD8^+^ T cells were then studied in adoptive transfer experiments (**Figure 4E**). 2x10^5^ purified peritoneal CD8^+^ T cells from PBS, vvDD or vvDD-IL15/Rα treated mice were given IP in conjunction with stimulatory doses of human IL-2 (50,000 IU) to 7-day peritoneal MC38-luc tumor-bearing mice after a preparatory regimen of sublethal whole-body irradiation (5Gy). Control mice received irradiation and IL-2 injections without T cell transfer. The results demonstrate an improved response and 55% long-term cures in the mice receiving T cells harvested from vvDD-IL15/Rα treated mice (**Figures 4F, G**).

Our approach should allow for ACT of solid tumors, even when naturally occurring specific antigens on tumor cells have not been identified yet. To proof our hypothesis, we examined the lavage CD8^+^ T cells from Balb/C mice bearing non-hypermutated, MMR-proficient CT26 colorectal cancer and B6 mice bearing MHCI-low expressing Panc02 pancreatic cancer. The peritoneal lavage CD8^+^ T cells from vvDD-IL15/Rα treated mice bearing CT26 (**Supplemental Figures 3A–D**) and Panc02 (**Supplemental Figures 3E–H**) demonstrated improved *in vitro* tumor-specific reactivity as measured by increased IFN-γ secretion (**Supplemental Figures 3B, F** respectively) and 4-1BB expression (**Supplemental Figures 3C, G** respectively) after coculture with specific target CT26 and Panc02 cells but not unspecific target cells 4T1 and MC38, respectively. In contrary peritoneal lavage vvDD-induced CD8^+^ T cells displayed unspecific reactivity with increased IFN-γ secretion after coculture with specific CT26 and Panc02 target cells and unspecific 4T1 and MC38 target cells (**Supplemental Figures 3B, F** respectively). Terminally exhausted phenotype (Tim-3^+^ PD-1^high^) CD8^+^ T cells were only slightly increased following vvDD-IL15/Rα treatment when cocultured with CT26 or Panc02, respectively, and were markedly increased after coculture with vvDD-infected tumor cells (**Supplemental Figures 3D, H**).

### Human Peritoneal CD8^+^ and CD4^+^ T Cells of Patients With Peritoneal Metastases Are Exhausted With Infrequent Levels of Tumor-Specific Recognition

**Figure 5:**
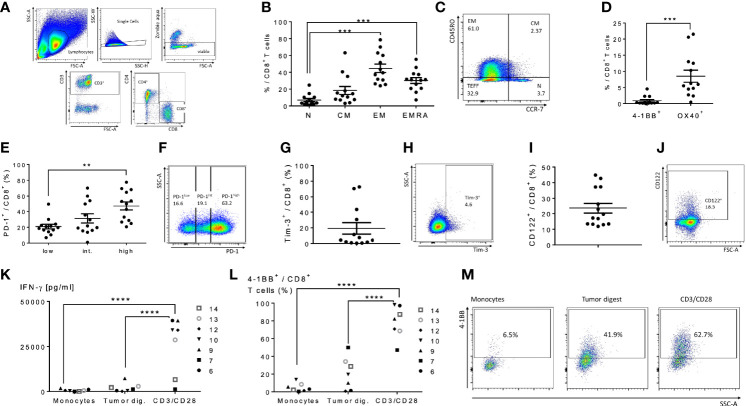
Characterization of human peritoneal CD8^+^ and CD4^+^ T cells derived from peritoneal metastasized tumors. Peritoneal fluid was collected from 14 patients undergoing peritoneal surgery, and peritoneal lymphocytes were extracted and characterized. **(A)** Flowchart of flow cytometry gating to identify peritoneal CD4 and CD8 T cells. Single cells gating, dead cells exclusion *via* Zombie aqua staining. Subsequently CD3^+^ cells were separated into CD4^+^ or CD8^+^ cells. **(B)** Distribution of naïve T cells (Tn, CD45RO^-^ CCR7^+^), central memory T cells (Tcm, CD45RO^+^ CCR7^+^), effector memory T cells (Tem, CD45RO^+^ CCR7^-^) and highly differentiated effector T cells (Temra, CD45RO^-^ CCR7^-^) gated from CD8^+^ T cells. **(C)** Representative flow cytometry chart of peritoneal memory CD8^+^ T cell subsets. **(D)** Levels of 4-1BB and Ox40 on CD8^+^ T cells were analyzed. **(E–H)** Analysis of PD-1 and Tim-3 demonstrated the majority of CD8^+^ cells were PD-1 high and an average of 20% expressed Tim-3. Representative flow charts of PD-1 and Tim-3 expression are shown in **(F, H)** respectively. **(I, J)** (representative flow chart): The shared beta-subunit of the IL-2- and IL15-receptor (CD122) showed a mean expression in peritoneal CD8^+^ T cells of 29.1%. **(K–M)** Peritoneal CD8^+^ T cells from 7 patients were tested for secretion of cytotoxic IFN-γ by ELISA **(K)** and 4-1BB surface expression by flow **(L, M)** (representative images) after overnight coculture with correlating tumor digest. Monocytes were used as negative controls, CD3/CD28 dynabeads as a positive control. All values presented as mean ± SEM. *p < 0.05. **p < 0.01. ***p < 0.001. ****p < 0.0001.

We next performed an analysis of peritoneal CD4^+^ and CD8^+^ T cells in cancer patients with peritoneal metastases to assess the feasibility of peritoneal lavage harvest for ACT in these patients as a correlate to our findings in murine models. We selected 14 patients from 36 to 75 years of age undergoing peritoneal cytoreductive surgery, including eight patients with appendiceal carcinomatosis, two patients with gastric carcinomatosis, two with colorectal cancer, one ovarian cancer and one mesothelioma (**Table 1**). Seventy percent received neoadjuvant chemotherapy prior to surgery. Cells were harvested from suction catheters following standard irrigation of the abdominal cavity. Total counts of recovered T cells are listed in **Table 1**, and ranged up to 1.3x10^8^ with roughly an equal number of CD4^+^ and CD8^+^ cells. CD8^+^ T cells were primarily of EM, effector memory, and EMRA, recently activated effector memory, phenotypes (**Figures 5B, C**). They expressed low levels of CD137/4-1BB and CD134/OX40 (**Figure 5D**), high PD-1 (**Figures 5E, F**), and greater than 10% Tim-3 (**Figures 5G, H**). The cells had high levels of CD122 (the β-chain of IL-2/IL-15 receptor), suggesting they would respond favorably to vvDD-IL15/Rα (**Figures 5I, J**). Analysis of CD4^+^ T cells demonstrated similar results (**Supplemental Figures 4A–I**). In seven patients we were able to assess *in vitro* responses to autologous tumor targets following coculture (**Figures 5K–M**). T cells from three patients showed significantly increased IFN-γ secretion after coculture with tumor digest (**Supplemental Figures 4J–L**), suggesting some tumor specific T cell reactivity. Cumulative analysis, however, found that IFN- γ secretion and 4-1BB surface expression were not significantly upregulated following coculture with autologous tumor digests (**Figures 5K, L**).

**Table 1:** Characteristics and T cell yield of patients with peritoneal metastases characteristics.

#	Age Range	Histological Type of primary tumor	Neoadjuvant therapy	T cell Recovery (x10^3^)	CD8^+^/CD4^+^ Recovery (x10^3^)
1	41–45	Appendiceal carcinomatosis; MACA-Signet cells-G3	FOLFIRINOX	4,158	1,638/2,024
2	46–50	Appendiceal carcinomatosis; MACA-Signet cells-G3	FOLFOX	3,600	1,487/1,691
3	56-60	Appendiceal carcinomatosis; MACA-Moderately differentiated-G2	FOLFIRINOX	3,956	1,685/2,017
4	71–75	Appendiceal carcinomatosis; MACA-Signet cells-G3	none	5,586	3,888/1,145
5	66–70	Colon cancer carcinomatosis; non-mucinous, moderately differentiated	FOLFOX	2,048	1,065/840
6	56–60	Appendiceal carcinomatosis; Goblet Cell Carcinoma, Tang C/G3	FOLFOX	11,104	5,974/4,086
7	61–65	Appendiceal carcinomatosis; MACA-signet cells-G3	FOLFOX	4,795	2,316/2,272
8	66–70	Appendiceal carcinomatosis; MACA-signet cells-G3	FOLFIRINOX	6,855	3,928/2,324
9	36–40	Ovarian carcinomatosis; low-grade serous neoplasm	none	13,000	4,953/7,397
10	51–55	Gastric carcinomatosis; poorly differentiated, diffuse type no signet cells present	FOLFOX	1,600	1,011/506
11	51–55	Appendiceal carcinomatosis; MACA-Signet cells-G3	none	6,000	4,290/1,380
12	51–55	Mesothelioma; epithelioid type	Cisplatin plus pemetrexed	10,680	5,073/5,128
13	46–50	Gastric carcinomatosis; poorly differentiated, diffuse type signet cells present	none	2,340	1,076/866
14	46–50	Colon cancer; Non mucinous, moderately differentiated	FOLFIRI + Avastin	6,271	3,831/2,050

MACA, Mucinous adenocarcinoma of the appendix; FOLFIRINOX, 5-FU, Leucovorin, Oxaliplatin, Irinotecan; FOLFOX, 5-FU, Leucovorin, Oxaliplatin; FOLFIRI, 5-FU, Leucovorin, Irinotecan.

We performed analyses on the effect of neoadjuvant therapy, age and histology on T cell phenotypes. Two out of the three individuals with autologous tumor responsiveness of T cells received neoadjuvant therapy, one of three individuals was diagnosed with appendiceal cancer and one of three individuals was older than 60 years. Our analysis revealed a great inter-tumoral and inter-individual heterogeneity in T cell phenotype. The number of patients examined was not high enough to reveal conclusive differences in T cell characteristics in these categories.

## Discussion

Cancer immunotherapy has been successful particularly in immune infiltrated tumors (28–30). Most solid tumors, however, are immune-excluded or immune-desert and not fully responsive to immunotherapy approaches (10, 31). Expanding the success of immunotherapy to these tumors represents one of the most critical challenges in the field of oncology research. Adaptive and acquired mechanisms of immunotherapy resistance (32) may include: 1) a lack of antigen presenting cells (33); 2) and of strong *de novo*-derived antigens; 3) expression of immune suppressive proteins (30); 4) down regulation of antigen presentation machinery (34); 5) reduced expression of immunogenic antigens (35); 6) recruitment of immune suppressive cells; 7) activation of the WNT-β-catenin pathway (36); 8) loss of the PTEN expression (37); 9) downregulation of T cell attracting chemokines (38); and 10) metabolic derangements (39). Because of the varied mechanisms of immune resistance, a single immunologic strategy or agent is unlikely to be successful.

TIL therapy has been studied for more than 35 years, resulting in durable tumor extinction after a single treatment if suitable tumor-specific T cells can be recovered (7). A recent review of TIL trials in melanoma reported an overall response rate of 42% (6). To date, most durable responses and survival with TIL has been observed in melanoma, an immunogenic tumor. A protocol for TIL therapy to treat uveal melanoma — a non-immunogenic form of melanoma arising in the eye and resistant to checkpoint inhibitor therapy – has demonstrated success (8). In this trial, TIL were selected based on autologous tumor-reactivity prior to re-infusion. The absence of autologous tumor-specific T cells in most solid tumors is a major limitation for TIL therapy.

Oncolytic virus immunotherapy has the unique ability to overcome the immune suppressive activity in the TME. The direct cytolytic effect of the virus results in diverse antigen, pro-inflammatory chemokine and cytokine release and drives IFN signaling to promote attraction and activation of immune cells (40). This potent, adaptive immune response leads to T cell mediated elimination of the virus, and in some cases the formation of anti-cancer adaptive immunity (2). The expression of immune transgenes by an OV can improve the anti-tumor immune response. The development of soluble IL-15 superagonist, greatly augmented the potency of the cytokine as a cancer immunotherapeutic agent (41). The combined immunotherapeutic potential of the IL-15 superagonist and the tumor selective vvDD enhanced survival of mice in IP models of MC38 colorectal and ID8 ovarian cancers (17), with CD8^+^ T cells being the key cells mediating vvDD-IL15/Rα antitumor therapeutic efficacy. *In vitro* stimulation of terminally exhausted CD8^+^ TIL from non-small-cell lung cancer patients with IL-15 rescued lymphocyte responsiveness to PD-1 blockade and enhanced proliferation (42).

Here, we have explored an ACT priming strategy to improve the number, phenotype and function of TIL using a model of peritoneal metastases – a common mode of spread of many solid tumors, associated with only limited therapeutic options. Infection of an aggressive MC38 peritoneal tumor with oncolytic vaccinia virus expressing IL-15/IL-15Rα leads to the recovery of a significantly increased number of cytotoxic T cells from peritoneal lavage samples that display an anti-tumor immune response and endogenous retroviral tumor antigen p15E-reactivity. These CD8^+^ T cells display a favorable memory phenotype, are not terminally exhausted, and can be re-directed for ACT. Treatment with vDD-IL15/Rα induced memory CD8^+^ T cells with anti-tumor immune responses not only in peritoneal carcinomatosis from MC38 colon cancer but also non-hypermutated CT26 colon and low MHC-1-expressing Panc02 pancreatic cancers (17). While the validity of the MC38 tumor model as an immune-desert tumor is debatable, the additional examination of low MHC-1-expressing Panc02 pancreatic cancers and the non-hypermutated CT-26 colon tumors heightens the possibility that these findings can be translated to human tumors. In addition, the control PBS treated MC38-bearing mice did not have any significant numbers of T-cells recovered on day 9 after inoculation, suggesting strong immunosuppression. The late-stage MC38 tumor model has previously been associated with increased immunosuppressive factor expression in the tumor microenvironment similar to human tumors (16).

It is important to note that the control animals had undetectable p15E-specific CD8^+^ T cells in the peritoneal lavage fluid throughout the study period of 14 days, whereas vvDD-IL15/Rα infection led to almost 1.25 x 10^5^ p15E positive cells when examined eight days following infection. p15E is an endogenous retroviral coat protein sequence present in the genome and expressed only in malignant tumors in B6 mice (26, 43). This tumor-rejection antigen encoded by endogenous retroviruses is also expressed in human tumors (43) and while it may be hard to identify T cells specific for these epitopes, after vvDD-IL15/Rα infection these T cells may be abundant and applicable for ACT.

One concern of using OV to prime T cells was whether a problematic dominance of anti-viral lymphocytes would limit emergence of any anti-tumor lymphocytes. Our data suggests the opposite for vvDD-IL15/Rα. In fact, while anti-viral T cells were slightly more prevalent in the beginning of the immune response, a higher percentage of p15E- and tumor-specific cells than B8R- or virus-specific cells were present over time only in the vvDD-IL15/Rα treatment group coinciding with viral clearance. This suggests that IL-15/IL-15Rα expression had a differential effect on CD8^+^ T cells recognizing tumor. It may be that T cells responding to the p15E tumor antigen have higher IL-15 receptors leading to a better proliferative response. On the other hand, stimulation of the anti-viral immune response by IL-15 superagonist accelerates viral clearance, which may alleviate the adaptive immune response of the T cell population. In addition, our previous work corroborates the swifter clearance of vaccinia virus accumulation in tumor cells by vvDD-IL15/Rα than vvDD (17), which may further shift the equilibrium of T cell recognition toward cancer antigens. In any case, it provides a window where the recovered T cell population demonstrated a strong anti-tumor response, ideal for ACT.

Additional techniques exist that have not been examined in this study to potentially improve TIL therapy including combination with other cytokine-armed OVs, pro-inflammatory cytokines such as IL-2 or IL-15 without a vector, checkpoint inhibitors, TLR9 agonists, CBL-B inhibitors and others. The synergy of oncolytic virotherapy and other combinational immunotherapies has also been established for adoptive T cells expressing chimeric antigen receptors (CART)-cell therapy. The oncolytic effects of EGFR-targeting, bispecific T-cell engager expressing oncolytic adenovirus on the immunosuppressive TME have been shown to improve activation and proliferation of CART cells *in vitro* (44). Solid tumors challenge CART-cell therapies by exhibiting tumor-antigen heterogeneity and by restricting tumor cell killing through CAR-targeted antigen loss. Tumor antigen-armed oncolytic virus can potentially overcome this mechanism of tumor escape by redirecting untransduced (CAR^-^) T cells toward secondary tumor antigens (44). We also show that the effector potential of endogenous intraperitoneal T cells can be exploited and redirected toward tumor antigens by IL15/Rα-armed oncolytic vaccinia virus. These IL15/Rα-primed endogenous intraperitoneal CD8^+^ T cells exhibited increased activation and killing activity *in vitro* and improved survival of adoptive cell therapy *in vivo*.

In addition to neglecting combinational immunotherapies in our study, we have primarily focused on *in vivo* enhancement of CD8^+^ T cells, as this cell type is most commonly applied and reactive in ACT (45). By focusing on CD8^+^ T cells, we were allowed more in-depth analysis of the phenotype for the purpose of more extensive and detailed functional examination. ACT with CD4^+^ T cells and other cell types including NK-cells, macrophages and dendritic cells are also being explored (45, 46). We have previously examined IL-2 expression by vvDD in a subcutaneous model and demonstrated feasibility of TIL therapy in that model (11). Successful ACT requires long-term maintenance of transferred cells, which depends on the presence and persistence of memory T cells. IL-15 clearly enhances memory phenotype and our results were consistent with that interpretation (47). While it was felt beyond the scope of this paper, further studies will explore the ability of vvDD-IL15/Rα to induce tumor reactive T-cells using *ex vivo* human models.

The feasibility of redirecting peritoneal lavage harvested T cells for ACT was demonstrated with our patient samples. Patients with ovarian, gastric, pancreatic, small bowel, colorectal, gallbladder, appendiceal, and mesothelioma cancers commonly develop peritoneal metastases (accounting for hundreds of thousands of patients world-wide), and a simple method for harvesting T cells would be peritoneal lavage (48). We have demonstrated here that peritoneal T cells display tumor-specific activation and cytotoxicity *in vitro* as well *as in vivo* (17), and equated the peritoneal macro-environment to the tumor microenvironment in models of peritoneal metastases. Moreover, we were able to recover peritoneal T cells following peritoneal irrigation in patients with peritoneal metastases. These T cells expressed the IL-15Rβ chain but lacked demonstrable autologous tumor reactivity, as is usually the case with gastrointestinal TIL, while displaying an exhausted phenotype.

IP delivery of OV *via* an indwelling catheter has previously been performed in clinical trials (49), could be safely performed as an out-patient and repeated as necessary. A major concern for harvesting T cells following vaccinia infection, would be the risk of recovering live virus with the specimen, or reactivation of vaccinia infection during the period of non-myeloablative chemotherapy utilized as a component of the TIL regimen. Our previous patient trials with vvDD demonstrated safety with up to 3x10^9^ pfu of vvDD delivered intravenously. The virus was cleared within a few days (50, 51). The expression of IL-15/IL-15Rα leads to earlier clearance of the virus in animal models. The optimal time for harvest seems to be 9-14 days after viral infection in animal models, which is beyond the ability to recover live virus with the specimen. As we and many other institutions are currently performing autologous TIL trials, these studies would serve as a favorable platform to evaluate the additional step of injecting vvDD-IL15/Rα into the tumor or IP 10 days prior to lavage or surgery. Administration of vvDD-IL15/Rα immunotherapy to significantly improve the quantity and quality of tumor-specific T cells, reactive against a diverse arrangement of tumor antigens for *ex vivo* expansion and autologous transfer would represent a critical advancement in this therapeutic strategy, and greatly expand the number of patients responsive to TIL therapy.

## Data Availability Statement

The original contributions presented in the study are included in the article/**Supplementary Material**. Further inquiries can be directed to the corresponding authors.

## Ethics Statement

The studies involving human participants were reviewed and approved by IRB of the University of Pittsburgh. The patients/participants provided their written informed consent to participate in this study. The animal study was reviewed and approved by Institutional Animal Care and Use Committee of the University of Pittsburgh.

## Author Contributions

EG, HK, ZL, MF, and CM conducted experiments and collected data. EG, ZG, and DB designed the project and wrote the manuscript. UK and ML provided expert advice on adoptive T cell transfer and immunology. All authors contributed to the article and approved the submitted version.

## Funding

EG and MF were supported in part by German Research Foundation (DFG research fellowships 1408/1-1 and 1655/1-1, respectively). This work was supported in part by The UPMC Immune Transplant and Therapy Center. This project used University of Pittsburgh shared core facilities (including the Animal Facility, Genomics Research Core, and Flow Cytometry and Small Animal Imaging services) supported in part by NIH award P30CA047904.

## Conflict of Interest

Author HK was employed by Kyowa Kirin Co., Ltd. in Japan. The authors DB, ZL, ZG, and MF have filed a patent application (US application number 62/454,526) which covers part of the approach described in this manuscript.

The remaining authors declare that the research was conducted in the absence of any commercial or financial relationships that could be construed as a potential conflict of interest.

## Supplementary Material

Supplementary Figure 1 vvDD-IL15/Rα treatment promotes an activated T cell response **(A–C)**: Animals from **Figure 1** were also analyzed for PD-1, PD-L1, and CTLA4 expression. 3 days after IP treatment, tumor tissue was harvested, total RNA isolated and transcribed into cDNA to be used for qPCR analysis. Relative mRNA expression levels of vvDD and vvDD-IL15/Rα were normalized to PBS treatment. Data are combined from two independent experiments. PD-1 (PDCD1) mRNA **(A)**, PD-L1 mRNA **(B)** and CTLA-4 mRNA **(C)** levels showed elevated expression in tumor tissue after vvDD and vvDD-IL15/Rα treatment in comparison to PBS. D: Tumor tissue from MC38-tumor bearing mice 3, 6 and 10 days after i.p. oncolytic virotherapy was analyzed for the vaccinia virus A34R gene, a marker of vaccinia virus accumulation. Gene expression indicates vvDD and vvDD-IL15/Rα replication on day 3 and partial vvDD replication on day 6 following i.p. treatment. All values presented as mean ± SEM. *p < 0.05. **p < 0.01. ***p < 0.001. ****p < 0.0001.

Supplementary Figure 2 Representative flow cytometry charts of immune checkpoint PD-1 surface expression by lavage and tumor infiltrating CD8^+^ T cells 10 days after oncolytic virotherapy. Representative flow cytometry charts of lavage and tumor infiltrating T cells from MC38 tumor bearing mice are shown. 10 days following i.p. oncolytic virotherapy with vvDD-IL15/Rα of late-stage MC38 tumor bearing mice, peritoneal CD8^+^ T cells were analyzed by FACS for PD-1 surface expression. **(A–C)**: Representative charts of tumor infiltrating CD8^+^ T cells from PBS-, vvDD- and vvDD-IL15/Rα-treated MC38 tumor bearing mice. **(D–F)**: Representative charts of lavage CD8^+^ T cells from PBS-, vvDD- and vvDD-IL15/Rα-treated MC38 tumor bearing mice; visualizing **Figure 2J**. PD-1^low^ CD8^+^ T cells are equivalent to PD-1^-^ CD8^+^ T cells.

Supplementary Figure 3 vvDD-IL15/Rα treatment enriches autologous tumor-specific CD8+ T cells in Panc02 pancreatic and CT26 colorectal carcinoma models of low MHC-1-expressing and non-hypermutated cancers, respectively. **(A–D)**: 6-8 week old Balb/C mice were IP inoculated with 1.5x10^6^ CT26 tumor cells, randomized 9 days later and IP injected with 5x10^5^ pfu oncolytic virus **(A)**. 14 days after vvDD-IL15/Rα treatment, peritoneal CD8^+^ T cells secrete cytotoxic IFN-γ and express costimulatory molecules 4-1BB **(B, C)** when cocultured *in vitro* with specific autologous CT26 and vvDD-infected 4T1 mammary carcinoma cells (inf. 4T1). vvDD-induced peritoneal CD8^+^ T cells only display viral reactivity. Exhausted Tim-3^+^ PD-1^high^ CD8^+^ T cells are significantly increased after coculture with vvDD-infected 4T1 cells 14 days after vvDD or vvDD-IL15/Rα treatment **(D)**. **(E–H)**: 6-8 weeks old B6 mice were IP inoculated with 1.5x10^6^ Panc02 pancreatic carcinoma cells, randomized 9 days later and IP injected with 5x10^7^ pfu oncolytic virus **(E)**. 14 days after vvDD-IL15/Rα treatment, peritoneal CD8^+^ T cells secrete cytotoxic IFN-γ and express costimulatory molecules 4-1BB **(F, G)** when cocultured *in vitro* with specific autologous Panc02 and vvDD-infected MC38 cells. vvDD-induced peritoneal CD8^+^ T cells only display viral reactivity. Exhausted Tim-3^+^ PD-1^high^ CD8^+^ T cells are significantly increased after coculture with vvDD-infected MC38 cells **(H)**. All values presented as mean ± SEM. *p < 0.05. **p < 0.01. ***p < 0.001. ****p < 0.0001.

Supplementary Figure 4 Characterization of peritoneal CD4^+^ T cells from patients with peritoneal carcinomatosis. As in **Figure 5**, peritoneal fluid was collected from 14 patients undergoing peritoneal surgery, and peritoneal lymphocytes were extracted. To detect lymphocyte subsets, mononuclear cells were isolated by gradient centrifugation. After two washes with PBS (phosphate buffered saline), isolated cells were characterized. **A–F**: CD4^+^ cells were phenotypically characterized by FACS for surface expression of memory markers CCR7 and CD45RO (A with representative chart in **B**), checkpoint markers PD-1 (**C**, representative chart in D), IL15 receptor beta CD122 (E with representative chart in **F**), and Tim-3 **(G, H)** and costimulatory molecules 4-1BB/CD137 and OX40/CD134 **(I)**. **J–L**: The CD8^+^ T cells from 3 individual patients (#7, 9 and 14) were characterized for autologous tumor responsiveness by IFN-γ production after coculture with tumor digest, CD3/CD28 positive control, and autologous peripheral blood monocytes for a negative control. These 3 patients demonstrated some autologous tumor reactivity.
