# Supplementary figures and images for: In Vivo Priming of Peritoneal Tumor-Reactive Lymphocytes With a Potent Oncolytic Virus for Adoptive Cell Therapy
Source: Front Immunol. 2021 Feb 18;12:610042. doi: 10.3389/fimmu.2021.610042 (PMC7930493; doi:10.3389/fimmu.2021.610042)

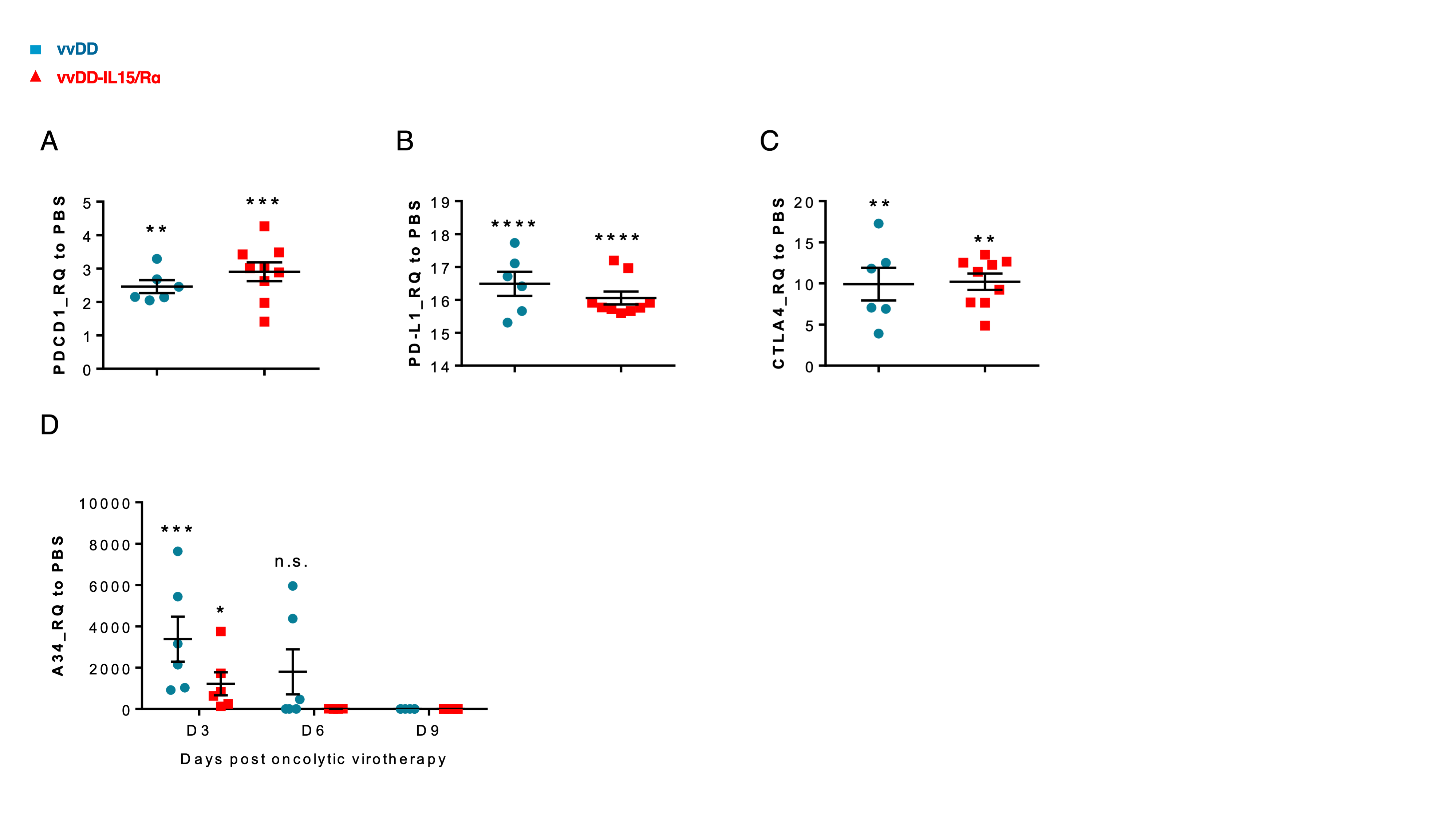

Supplement: Supplementary Figure 1 — vvDD-IL15/Rα treatment promotes an activated T cell response (A–C): Animals from Figure 1 were also analyzed for PD-1, PD-L1, and CTLA4 expression. 3 days after IP treatment, tumor tissue was harvested, total RNA isolated and transcribed into cDNA to be used for qPCR analysis. Relative mRNA expression levels of vvDD and vvDD-IL15/Rα were normalized to PBS treatment. Data are combined from two independent experiments. PD-1 (PDCD1) mRNA (A), PD-L1 mRNA (B) and CTLA-4 mRNA (C) levels showed elevated expression in tumor tissue after vvDD and vvDD-IL15/Rα treatment in comparison to PBS. D: Tumor tissue from MC38-tumor bearing mice 3, 6 and 10 days after i.p. oncolytic virotherapy was analyzed for the vaccinia virus A34R gene, a marker of vaccinia virus accumulation. Gene expression indicates vvDD and vvDD-IL15/Rα replication on day 3 and partial vvDD replication on day 6 following i.p. treatment. All values presented as mean ± SEM. *p < 0.05. **p < 0.01. ***p < 0.001. ****p < 0.0001. [file DataSheet_1.zip › Supplementary Figure 1.TIFF]

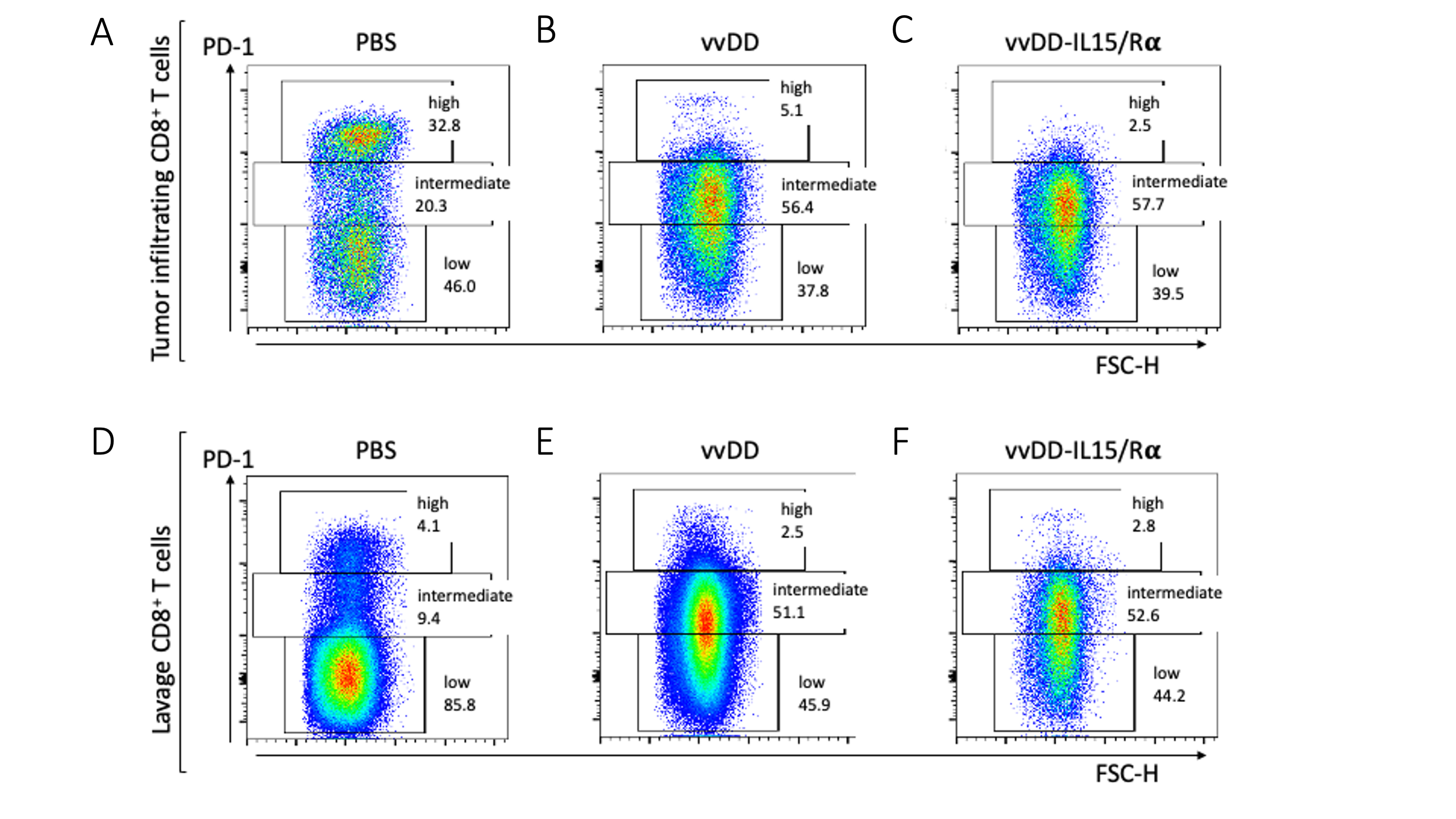

Supplement: Supplementary Figure 1 — vvDD-IL15/Rα treatment promotes an activated T cell response (A–C): Animals from Figure 1 were also analyzed for PD-1, PD-L1, and CTLA4 expression. 3 days after IP treatment, tumor tissue was harvested, total RNA isolated and transcribed into cDNA to be used for qPCR analysis. Relative mRNA expression levels of vvDD and vvDD-IL15/Rα were normalized to PBS treatment. Data are combined from two independent experiments. PD-1 (PDCD1) mRNA (A), PD-L1 mRNA (B) and CTLA-4 mRNA (C) levels showed elevated expression in tumor tissue after vvDD and vvDD-IL15/Rα treatment in comparison to PBS. D: Tumor tissue from MC38-tumor bearing mice 3, 6 and 10 days after i.p. oncolytic virotherapy was analyzed for the vaccinia virus A34R gene, a marker of vaccinia virus accumulation. Gene expression indicates vvDD and vvDD-IL15/Rα replication on day 3 and partial vvDD replication on day 6 following i.p. treatment. All values presented as mean ± SEM. *p < 0.05. **p < 0.01. ***p < 0.001. ****p < 0.0001. [file DataSheet_1.zip › Supplementary Figure 2.TIFF]

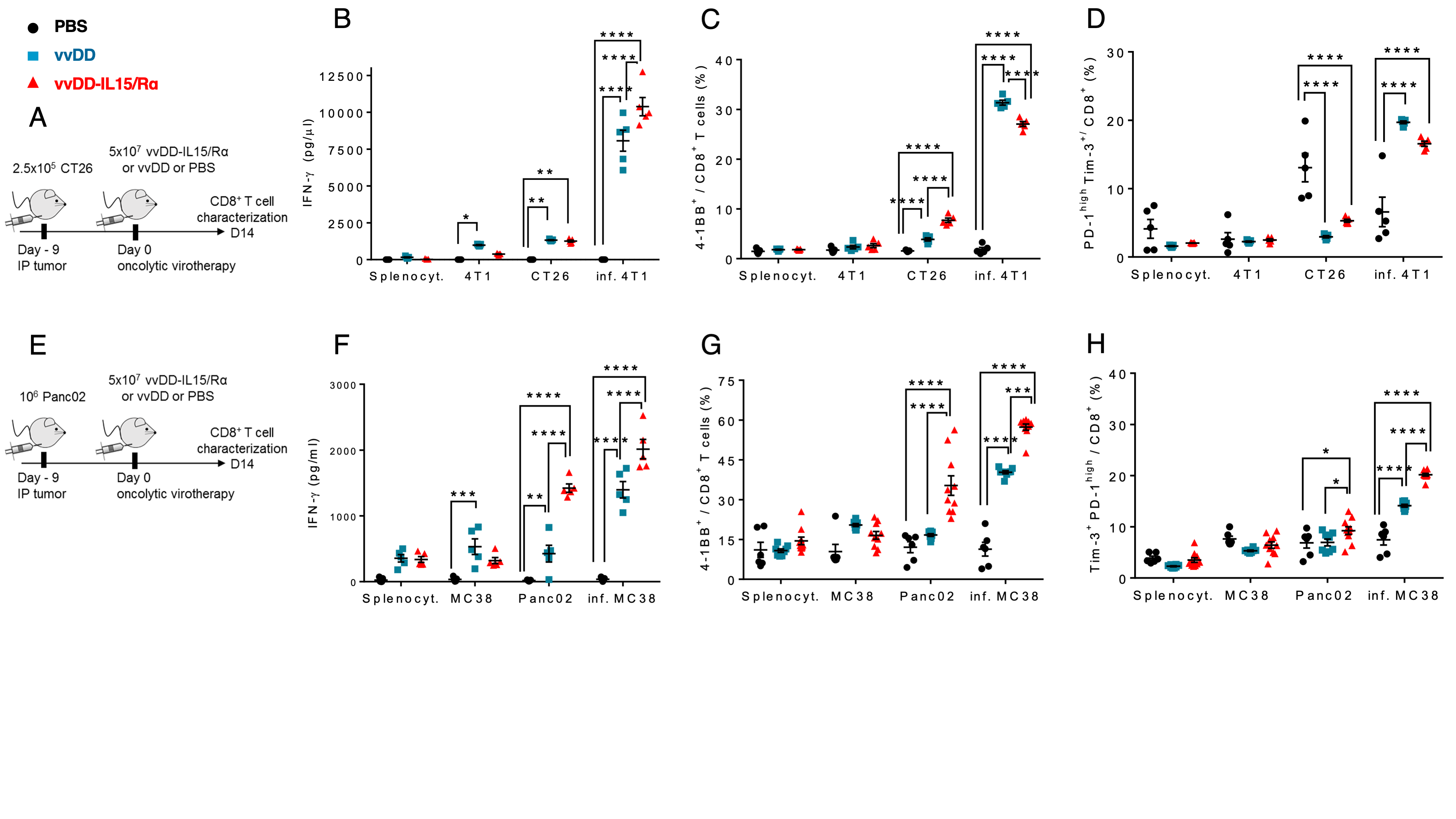

Supplement: Supplementary Figure 1 — vvDD-IL15/Rα treatment promotes an activated T cell response (A–C): Animals from Figure 1 were also analyzed for PD-1, PD-L1, and CTLA4 expression. 3 days after IP treatment, tumor tissue was harvested, total RNA isolated and transcribed into cDNA to be used for qPCR analysis. Relative mRNA expression levels of vvDD and vvDD-IL15/Rα were normalized to PBS treatment. Data are combined from two independent experiments. PD-1 (PDCD1) mRNA (A), PD-L1 mRNA (B) and CTLA-4 mRNA (C) levels showed elevated expression in tumor tissue after vvDD and vvDD-IL15/Rα treatment in comparison to PBS. D: Tumor tissue from MC38-tumor bearing mice 3, 6 and 10 days after i.p. oncolytic virotherapy was analyzed for the vaccinia virus A34R gene, a marker of vaccinia virus accumulation. Gene expression indicates vvDD and vvDD-IL15/Rα replication on day 3 and partial vvDD replication on day 6 following i.p. treatment. All values presented as mean ± SEM. *p < 0.05. **p < 0.01. ***p < 0.001. ****p < 0.0001. [file DataSheet_1.zip › Supplementary Figure 3.TIFF]

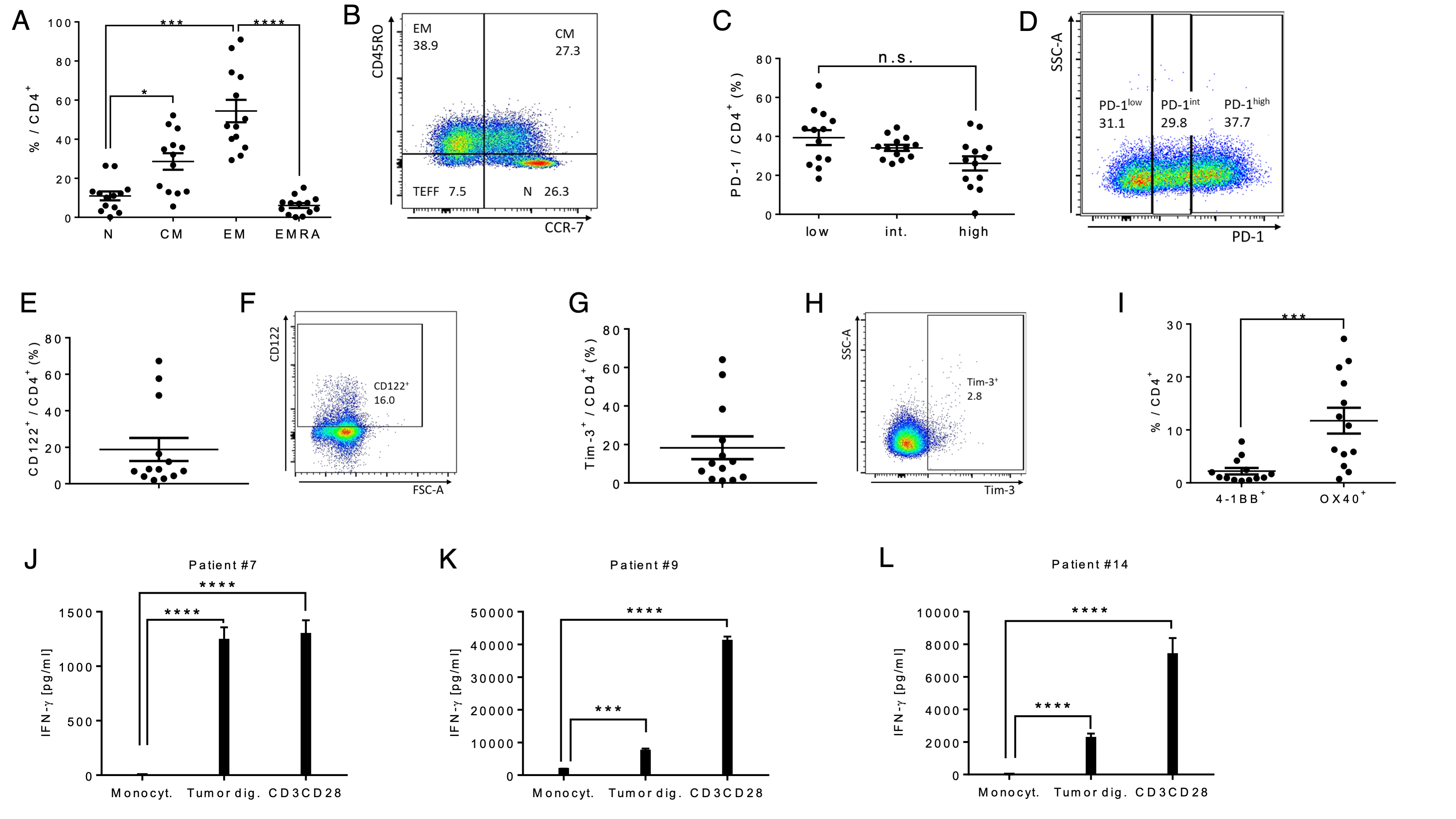

Supplement: Supplementary Figure 1 — vvDD-IL15/Rα treatment promotes an activated T cell response (A–C): Animals from Figure 1 were also analyzed for PD-1, PD-L1, and CTLA4 expression. 3 days after IP treatment, tumor tissue was harvested, total RNA isolated and transcribed into cDNA to be used for qPCR analysis. Relative mRNA expression levels of vvDD and vvDD-IL15/Rα were normalized to PBS treatment. Data are combined from two independent experiments. PD-1 (PDCD1) mRNA (A), PD-L1 mRNA (B) and CTLA-4 mRNA (C) levels showed elevated expression in tumor tissue after vvDD and vvDD-IL15/Rα treatment in comparison to PBS. D: Tumor tissue from MC38-tumor bearing mice 3, 6 and 10 days after i.p. oncolytic virotherapy was analyzed for the vaccinia virus A34R gene, a marker of vaccinia virus accumulation. Gene expression indicates vvDD and vvDD-IL15/Rα replication on day 3 and partial vvDD replication on day 6 following i.p. treatment. All values presented as mean ± SEM. *p < 0.05. **p < 0.01. ***p < 0.001. ****p < 0.0001. [file DataSheet_1.zip › Supplementary Figure 4.TIFF]
